# Catatonia - a Rare Manifestation of Wilson’s Disease

**DOI:** 10.1192/bjo.2026.11783

**Published:** 2026-06-30

**Authors:** Georgia Hasell, Moheb Gaid, Sameep Karkhanis

**Affiliations:** 1 Colman Centre for Specialist Rehabilitation Services, Norfolk Community Health and Care NHS Trust, Norwich, United Kingdom; 2 Norfolk and Norwich University Hospitals NHS Foundation Trust, Norwich, United Kingdom

## Abstract

**Aims:**

This case report reviews a rare presentation of a young male patient presenting with Catatonia, who was later diagnosed with Wilson’s Disease (WD), and explores the diagnostic challenges faced with atypical neuropsychiatric presentations and how this affects long-term outcomes.

**Methods:**

A previously healthy Caucasian man in his early-30’s presented with acute-onset catatonia over 48 hours, characterised by social withdrawal, psychomotor retardation, staring spells, and posturing. He was initially diagnosed as having catatonia with depression and psychotic symptoms and treated with Lorazepam, Sertraline, Promethazine, and Olanzapine. Over the proceeding few months he deteriorated, showing signs of tremulousness, muscle rigidity with autonomic features, and hyper-reflexia. MRI Head demonstrated abnormal signal intensity in the corpus striatum, ventrolateral thalami, and brainstem, with basal ganglia and substantia nigra hypointensity on SWI sequences suggestive of iron or mineral deposition. Blood tests revealed low caeruloplasmin (0.1g/L), elevated 24-hour urinary copper excretion (5.37umol/24h), elevated CK (1907 U/L), and elevated ALT (105U/L). Kayser-Fleischer Rings were evident on ophthalmology assessment, Liver Ultrasound revealed a coarse irregular liver, and genetic studies confirmed mutations in the ATP7B gene. He had no family history of liver or movement disorders. He was treated with penicillamine, zinc supplements, muscle relaxants, and anticholinergics with no paradoxical worsening.

**Results:**

Wilson’s Disease (WD) is a rare inherited autosomal recessive disorder of copper metabolism resulting mainly in neurological and hepatic pathology. However, in 10-25% it can present with progressive neuropsychiatric symptoms, often resulting in delayed diagnosis for up to 65-months. Notable psychiatric manifestations include personality and mood disorders, sleep disturbance, cognitive impairment, and occasionally psychosis. Rarely, WD can present with Catatonia – a neuropsychiatric syndrome characterised by mutism, stupor, posturing and catalepsy, echolalia and staring. Research suggests that up to 65.6% of patients with WD are initially misdiagnosed, prolonging commencement of treatment and potentially worsening patient outcome. Copper chelation therapy with D-Penicillamine and Zinc can in some cases reverse neuropsychiatric manifestations in WD, and if these are not tolerated Trientine Dihydrochloride is a possible second line agent.

**Conclusion:**

Wilson’s Disease (WD) is a rare condition with a vast range of clinical manifestations, and prompt diagnosis can improve patient outcomes. This case highlights the importance of having a high index of suspicion for WD, particularly during refractory acute-onset psychiatric presentations, and notably for those with no personal or familial psychiatric history. Diagnostic tools include testing for caeruloplasmin levels, 24-hour urinary copper excretion, ophthalmology assessment, MRI Head scanning, and ATP7B genetic testing for diagnostic confirmation.

